# Recent topics related to etiology and clinical manifestations of cutaneous arteritis

**DOI:** 10.3389/fmed.2022.1022512

**Published:** 2022-10-10

**Authors:** Takaharu Ikeda

**Affiliations:** Division of Dermatology, Faculty of Medicine, Tohoku Medical and Pharmaceutical University, Sendai, Japan

**Keywords:** anti-lysosomal-associated membrane protein-2 antibody, anti-phosphatidylserine-prothrombin complex antibody, cutaneous arteritis, deficiency of adenosine deaminase 2, livedo racemosa, polyarteritis nodosa

## Abstract

Cutaneous polyarteritis nodosa (cPAN) was first reported by Lindberg in 1931. It has been recognized as a skin-limited vasculitis whose cutaneous histopathological features are indistinguishable from those of PAN. Cutaneous arteritis (CA) was defined as a form of single-organ vasculitis in the revised Chapel Hill Classification and was recognized as the same disease as cPAN. It became known that deficiency of adenosine deaminase 2 (DADA2) cases were included in cases that had been diagnosed with CA. Because of their similarity and differences in the treatment methods, DADA2 should be considered in CA cases, especially if they are diagnosed or developed in early childhood. Cutaneous arteritis may be classified as an immune complex-mediated vasculitis. It was reported that the pathogenesis of anti-lysosomal-associated membrane protein-2 (LAMP-2) antibodies and anti-phosphatidylserine-prothrombin complex (PS/PT) antibodies as good parameters in CA. The main skin manifestations include livedo racemosa, subcutaneous nodules, and ulcers. Although CA is recognized to have a benign clinical course, it has become known that it is easy to relapse. The existence of skin ulcers upon diagnosis or sensory neuropathies was suggested to be a predictor of poor prognosis. Cutaneous arteritis with them may need to be treated with more intensive therapies.

## Introduction

Polyarteritis nodosa (PAN) is a systemic necrotizing vasculitis that involves medium-sized muscular arteries as well as small-sized muscular arteries without glomerulonephritis. It can form segmental lesions in the damaged vessels and can affect multiple organs, such as the skin, nerves, and kidneys.

Kussmaul and Maier (1) have reported inflammatory arterial nodules as periarteritis nodosa. It was then revealed that the distribution of inflammation was observed in the whole arterial wall rather than the periarterial area, which was then renamed as PAN. Anti-neutrophil cytoplasmic antibodies (ANCA) were identified in cases with glomerulonephritis in 1982 (2) and in cases with microscopic polyangiitis in 1985 (3), and their pathogenicity was proven. Moreover, a group of small vessel vasculitides, such as microscopic polyangiitis, granulomatosis with polyangiitis, and eosinophilic granulomatosis with polyangiitis in which ANCA was involved in the pathogenesis, were classified as separate diseases from PAN. According to the revised Chapel Hill Classification (4) published by the Chapel Hill Consensus Conference in 2012, PAN is defined as a necrotizing arteritis of medium or small arteries without glomerulonephritis or vasculitis in the arterioles, capillaries, or venules, that is not associated with ANCA. It is also considered as a rare disease.

Cutaneous polyarteritis nodosa (cPAN) was first reported as a skin variant of periarteritis nodosa by Lindberg in 1931 (5). It has been recognized as a skin-limited vasculitis without any other organ manifestations whose cutaneous histopathological features are indistinguishable from those of PAN. It affects medium-sized arteries at the dermal-subcutis junction and subcutis and small-sized arteries, and often shows subcutaneous nodules and livedo racemosa. The revised Chapel Hill Classification (4) classified the category of single-organ vasculitis, which affects arteries or veins of any size in a single organ and is not a limited expression of another systemic vasculitis. Cutaneous arteritis (CA) was described as a form of single-organ vasculitis and was recognized as the same disease as cPAN.

As mentioned in the classification, it was reported that cases with CA progressed into PAN (6, 7). This phenomenon is thought to be very rare, but cases with CA occasionally show general symptoms, such as musculoskeletal and peripheral neurologic manifestations within the distribution of skin lesions. As such cases can be diagnosed with PAN, Nakamura et al. proposed a diagnostic criteria for cPAN (8).

Cutaneous arteritis usually has a chronic and favorable clinical course, but occasionally relapses and needs to be treated with more intensive therapies.

## Epidemiology

Polyarteritis nodosa has been considered a rare disease due to the changes in the disease concept of PAN and the reduced onset of hepatitis B virus infection attributed to hepatitis B virus vaccination, with an estimated prevalence of 30.7 per million (9). On the contrary, CA is a rarer disease, and its prevalence has not been determined yet. Although it is known that the peak of the onset of PAN is in the sixth decade of life and that PAN predominantly affects females, CA develops commonly in the fourth and fifth decades of life and predominantly affects males. Recent cohort studies reported a female-to-male ratio of 1.22–3.53 and that the mean or median age of onset was around the fourth decade of life (Table 1) (7, 10–15). Like PAN, CA also affects children.

**Table 1:** Summary of previous reports on clinical manifestation.

	**Daoud**	**Kawakami**	**Criado**	**Alibaz-Oner**	**Ikeda**	**Munera-Campos**	**Bettuzzi**
	**1997**	**2013**	**2016**	**2017**	**2020**	**2020**	**2022**
	**USA**	**Japan**	**Brazil**	**USA**	**Japan**	**Spain**	**French**
Number of cases	79	101	22	41	84	31	68
Females/males	1.72	2.37	3.40	1.92	2.36	1.82	3.53
Mean age	—	45.4 ± 17.9	39.4 ± 15.2	49.1 ± 18.8	45.7 ± 15.3	47.5^*^	39^*^ (as the median)
(range)	(6–81)	(—)	(9–61)	(—)	(—)	(18–76)	(—)
**Distribution of skin lesions**							
Lower limbs	Legs 97.5%	Legs 100.0%	100.0%	—	Lower legs 84.5%	Lower legs 100.0%	100.0%
					Thighs 6.0%	Thighs 41.9%	
Upper limbs	Arms 32.9%	—	27.3%	—	2.4%	32.3%	20.5%
Trunk	7.6%	—	27.3%	—	0.0%	19.4%	11.8%
**Skin symptoms**							
Livedo racemosa	—	83.2%	54.5%	—	60.7%	—	77.9% (as livedo)
Livedo reticularis	55.7%	—		39.0%	3.6%	45.2%	
Subcutaneous nodules	—	—	50.0%	—	23.8%	90.3%	—
Nodules	79.7%	100.0%		61.0%	—	—	69.1%
Ulcers	49.4%	48.5%	63.7%	14.6%	30.0%	35.5%	16.1%
Purpura	—	66.3%	27.3%	31.7%	40.5%	3.2%	17.6%
Edema	—	31.6%		12.8%	30.0%	—	—
		(as leg edema)		(as peripheral extremity edema)			
Erythema	—	—		—	73.8%	—	—
**Constitutional symptoms**							
Fever	—	—	9.1%	19.5%	14.3%	9.7%	11.8%
Asthenia	—	—	—	—	—	67.7%	33.8%
Weight loss	—	—	4.5%	5.0%	0%	6.5%	11.8%
**Extra-cutaneous symptoms**							
Arthralgia	—	66.3%	9.1%	41.5%	—	19.4%	36.8%
Arthritis	—	—	—	—	36.9%	—	2.9%
Myalgia	—	44.6%	—	—	21.4%	58.1%	—
Paresthesia	—	—	36.3%	—	—	45.2%	—
Peripheral neuropathy	—	—	—	2.4%	33.3%	—	—
Neurological sensory involvement	—	—	—	—	—	—	32.4%
Mono-neuritis multiplex	—	56.4%	22.7%	0%	—	29.0%	—

—, Not described.

* Cases older than 18 years of age were studied.

## Etiology

Classic PAN and CA are diseases with unknown etiology. Although the hepatitis B virus has already been considered pathogenetic, hepatitis B virus-associated PAN is still considered rare in Japan. Other reported pathogenetic factors for CA included infections, such as *Mycobacterium* infection, Group A *Streptococcus* infection, hepatitis C virus infection etc.; autoimmune diseases; and medications, such as minocycline.

It became known that cases of deficiency of adenosine deaminase 2 (DADA2) were included in cases that had been diagnosed with PAN or CA.

Adenosine deaminase 2 (ADA2) is an extracellular protein that is secreted by monocytes, macrophages, etc. It deaminates and converts adenosine into inosine and regulates the extracellular adenosine concentration.

Deficiency of adenosine deaminase 2 is a recessively inherited autoinflammatory disease caused by the biallelic pathogenic variants in the ADA2 gene on chromosome 22q11, wherein many mutations have been reported. Family history is also often negative. Although most DADA2 cases develop during infancy or early childhood, adult-onset cases have also been reported. The genotype–phenotype correlations were known (16), but differences in the phenotypes, such as the age of onset, severity, and symptoms, can be observed between and within families even if the mutation is common within the families (17, 18).

Deficiency of adenosine deaminase 2 has a broad clinical spectrum and is characterized by vasculitis, which causes strokes and cutaneous manifestations, such as livedo racemosa and livedo reticularis; hematologic abnormalities, such as pancytopenia and bone marrow failure; and immunological manifestations. Vasculitis, which is observed in DADA2, affects medium- and small-sized vessels with histopathologic features that are indistinguishable from those of PAN.

Deficiency of adenosine deaminase 2 was first described in 2014. It was found that PAN cases, most of whom were pediatric and familial cases, have been associated with recessive loss-of-function mutations of ADA2. They were characterized by livedo reticularis and early-onset cerebral infarcts (17). Simultaneously, another study group reported nine cases, including two siblings with PAN characterized by livedo racemosa, early-onset lacunar strokes, and other neurovascular manifestations, carried recessively inherited loss-of-function mutations of ADA2 (18). Moreover, Gibson et al. showed that 9 of 60 primary chronic pediatric vasculitis cases that had been registered in the Pediatric Vasculitis Initiative international study had DADA2 and that 5 of 16 cases that had been diagnosed with PAN were proven to be variants (19). Schnappauf et al. showed that 9 of 118 cases with PAN carried variants in ADA2, while 4 cases had biallelic variants that were pathogenic or likely pathogenic (20).

In a cohort study of 58 cases of DADA2, cutaneous involvement was the most prevalent symptom. Of the cases, 90% had a history of skin involvement, 74% had livedo racemosa, and 57% had nodules (21). The initial symptoms in infancy or early childhood may include livedo racemosa, and severe systemic vasculitis and strokes may occur during in childhood as the patients grow. Other manifestations, such as subcutaneous nodules, purpura, livedo, Raynaud's phenomenon, and skin ulcerations, can also be observed (22).

Zavialov et al. have reported that ADA2 promoted macrophage differentiation from monocytes and its proliferation (23). Zhou et al. also reported the reduction of the serum levels of ADA2 and ADA2-specific enzyme activity in cases with recessively inherited mutations in ADA2 and monocytes from these cases differentiation into proinflammatory M1 macrophages rather than into anti-inflammatory M2 macrophages (18). This can result in a hyper-inflammatory environment that damages the blood vessels (24). Carmona-Rivera et al. have reported that neutrophil extracellular trap (NET) formation mediated by extracellular adenosine was enhanced and macrophages that were stimulated by NETs produced tumor necrosis factor (TNF)-α as well as determined the pathological roles of neutrophils in DADA2 (24).

Tumor necrosis factor-α inhibitors are common treatments for the vasculitis phenotype of DADA2. They improve the symptoms due to inflammation and vasculitis and significantly prevent strokes (25). However, they have an insignificant effect on the symptoms of bone marrow failure or immunodeficiency (26). The 2021 American College of Rheumatology/Vasculitis Foundation guidelines for managing PAN strongly recommended the treatment with TNF-α inhibitors over corticosteroids alone in cases with the clinical manifestations of DADA2 (27). Because of the similarity between DADA2 and PAN or CA and the differences in the treatment methods, DADA2 should be considered in PAN or CA cases, especially if they are diagnosed or developed in early childhood.

Although vascular damage during viral replication in hepatitis B virus related PAN or ADA2 mutation in DADA2 indistinguishable from PAN has been suggested to involve the onset, the pathogenesis of classic PAN or CA remains unclear.

However, Diaz-Perez et al. have shown that a direct immunofluorescence study using the skin samples obtained by excision biopsies indicated the C3 deposition in the vessel walls in 4 of 10 cPAN cases that were not associated with hepatitis B virus and IgM in 6 of 10 cases (28). Kawakami et al. showed a direct immunofluorescence study indicating the deposition of C3 in 22 (66.7%) of 33 cPAN cases that were not associated with hepatitis B virus and IgM in 19 (57.6%) of 33 cases (29). Overall, these results suggest a complement activation in the vessel walls and CA may be classified as an immune complex-mediated vasculitis. It was also reported that some antibodies might be pathogenetic in PAN or CA.

Lysosomal-associated membrane protein-2 (LAMP-2) is a glycoprotein in membranes of lysosomes and intracellular vesicles within neutrophils and endothelial cells and is an antigen for minor ANCAs. Kawakami et al. showed that the serum levels of anti-LAMP-2 antibodies in cases with cPAN were significantly higher than those in cases with microscopic polyangiitis (30). Takeuchi et al. observed that the intraveneous injection of anti-LAMP-2 antibodies to premorbid env-pX rats, which were the model mice of PAN-like necrotizing vasculitis, induced the neutrophilic infiltration to cutaneous small vessels and allowed the detection of anti-LAMP-2 antibody-binding neutrophils (31). Li et al. also found that the serum LAMP-2 levels in PAN cases were significantly higher than those in ANCA-associated vasculitis cases and were correlated with the Birmingham Vasculitis Activity Score and hypersensitive C-reactive protein (32). These results confirmed the pathogenesis of anti-LAMP-2 antibodies in cutaneous vasculitis.

Anti-phosphatidylserine-prothrombin complex (PS/PT) antibodies have been found to be associated with the clinical manifestations of antiphospholipid syndrome. It was reported that serum anti-PS/PT IgM antibodies were detected in 81.3% of cPAN cases although they were not detected in healthy individuals, and their levels in cPAN cases were significantly higher than those in systemic lupus erythematosus or microscopic polyangiitis cases (33). The levels of anti-PS/PT IgM antibodies were significantly higher in cPAN cases with livedo racemosa than in those without it (29). It was also reported that the levels of anti-PS/PT antibodies in PAN cases with active skin manifestations showing necrotizing vasculitis decreased significantly after treatment (34). Moreover, Sánchez-Cubías et al. described that the levels of anti-PS/PT IgM antibodies in all cases with inactive PAN and those of anti-PS/PT IgG antibodies in all cases except one case with inactive PAN were negative (35). These results suggest that anti-PS/PT antibodies may be good parameters of PAN and CA.

Furthermore, Kawakami et al. reported that the serum anti-PS/PT IgM antibodies levels were higher in the group of cases of systemic vasculitis with skin involvements (three cases of IgA vasculitis, two cases of eosinophilic granulomatosis with polyangiitis, one case of microscopic polyangiitis, and one case of granulomatosis with polyangiitis) and one case of CA than those in the group of cases of systemic vasculitis without skin involvements (two cases of eosinophilic granulomatosis with polyangiitis, two cases of microscopic polyangiitis, one case of granulomatosis with polyangiitis, one case of rheumatoid vasculitis, and one case of PAN), but no significant difference was observed in the serum anti-PS/PT IgG antibody levels (36). These results suggest that serum anti-PS/PT IgM antibodies might be involved in the pathogenesis of cutaneous vasculitis. Okano et al. reported the overexpression of moesin in affected skin vessels and that the titer of serum anti-moesin antibodies in PAN cases with skin involvements due to necrotizing vasculitis is positively correlated with the Birmingham Vasculitis Activity Score results and the Vasculitis Damage Index (34).

## Clinical features

Table 1 summarizes the clinical features that were reported in previous articles (7, 10–15). Constitutional symptoms such as fever, fatigue, asthenia, and weight loss are shown in only a few CA cases, but their incidence is lower than those in PAN cases. In the cohort study by Alibaz-Oner et al. the incidence of weight loss or fatigue in PAN cases was significantly higher than that in CA cases (7). The cause of constitutional symptoms in CA cases is unknown but it was explained that they resulted not from systemic vasculitis but from inflammatory reactions or the distribution of local inflammatory mediators (7, 14).

The main skin manifestations include livedo, subcutaneous nodules, and ulcers. Previous studies have shown that their prevalence was as follows: livedo racemose, 60.7–83.2%; livedo reticularis, 3.6–55.7%; nodules or subcutaneous nodules, 23.8–100%; skin ulcers, 14.6–19.4%. In the cohort study of Alibaz-Oner et al. the incidence of nodules in PAN cases was significantly lower than that in CA cases (7). The cutaneous symptoms usually concentrate in the lower extremities, and occasionally in the upper extremities, and to a lesser extent may involve the trunk.

Livedo racemosa is a morphologic incomplete network or net-like pattern composed of interrupted rings and is recognized as the consequence of the persistent disruption of blood flow secondary to organic rather than functional disorders (14, 37). On the other hand, livedo reticularis presents a complete lace pattern with regular rings and can be secondary to either organic or functional disorders (14, 15). Munera-Campos et al. have observed atrophie blanche in 25.8% of the study participants (14), while Criado et al. in 45.4% (12). These manifestations are characteristic but not specific for CA, requiring differentiation from systemic vasculopathy or thrombosis due to antiphospholipid syndrome, systemic lupus erythematosus, livedo vasculopathy, etc.

Musculoskeletal and peripheral neurologic manifestations of CA are occasionally observed. These incidences still remain lower than those in PAN. Previous studies have reported an incidence of arthralgia of 9.1–66.3%; myalgia, 21.4–58.1%; paresthesia, 36.3–45.2%; and mononeuritis multiplex, 0–56.4%. In the cohort study of Alibaz-Oner et al. the incidence of peripheral neuropathy in PAN cases was significantly higher than that in CA cases (7). It was suggested that these were secondary to the deep and intense focal skin damages (12) and appear within the distribution of skin lesions. When extracutaneous symptoms are observed outside the range of skin symptoms, systemic vasculitis such as PAN should be considered.

Although CA is recognized to have a benign clinical course, it has become known that CA is easy to relapse. Munera-Campos et al. have observed that 54.8% of CA cases experienced relapses (14), while Alibaz-Oner et al. found that the 5-year cumulative relapse rate was 45.2% in CA cases and 9.6% in PAN cases (7). Bettuzzi et al. showed that 31% of CA cases achieved complete response after first-line therapies, but 63% of CA cases had relapsing/refractory course and received second-line treatments (15). Cases that received a second-line treatment presented fever, nodules, or sensory neuropathy more frequently than those that received no treatment or a single treatment. On the other hand, Munera-Campos et al. showed that CA cases with relapse had ulceration upon diagnosis significantly more frequently than those without relapse (14). Shirai et al. showed that the relapse rate of CA cases with skin ulcers or necrosis was significantly higher than that of CA cases without ulcer or necrosis or that of PAN cases (38). Colchicine, dapsone, or corticosteroids alone is often administered for the treatment of CA, probably because CA damages a single organ and is recognized as having a favorable prognosis. It was suggested that the high relapse rate of CA might be due to the trend that immunosuppressive therapies had not been used frequently (7). Cutaneous arteritis with ulceration or peripheral neuropathy may require an early add-on intensive therapy.

## Author contributions

The author confirms being the sole contributor of this work and has approved it for publication.

## Conflict of interest

The author declares that the research was conducted in the absence of any commercial or financial relationships that could be construed as a potential conflict of interest.

## Publisher's note

All claims expressed in this article are solely those of the authors and do not necessarily represent those of their affiliated organizations, or those of the publisher, the editors and the reviewers. Any product that may be evaluated in this article, or claim that may be made by its manufacturer, is not guaranteed or endorsed by the publisher.
